# Association of sleep duration with stroke, myocardial infarction, and tumors in a Chinese population with metabolic syndrome: a retrospective study

**DOI:** 10.1186/s12944-020-01328-1

**Published:** 2020-06-27

**Authors:** Yingnan YE, Linxi ZHANG, Anping WANG, Yuxia Wang, Shiqing WANG, Guang NING, Yiming MU

**Affiliations:** 1 Department of Endocrinology, Chest Hospital Affiliated to Nankai University, School of Medicine, Nankai University, Tianjin, 300071 China; 2 Department of Endocrinology, Chinese PLA General Hospital, Medical School of Chinese PLA, No.28, Fuxing Road, Haidian District, Beijing, 100853 China; 3 Department of Endocrine and Metabolic Diseases, Ruijin Hospital, Shanghai Jiao Tong University School of Medicine, 200025 Shanghai, China

**Keywords:** Sleep duration, Metabolic syndrome, Stroke, Myocardial infarction, Tumors, Cohort, Chinese population

## Abstract

**Background:**

Previous studies have suggested that abnormal sleep duration is associated with increased risk of metabolic syndrome (MetS). However, evidence on the association of sleep duration with stroke, myocardial infarction (MI) and tumors in populations with MetS is limited.

**Methods:**

A total of 8968 participants (2754 with MetS at baseline) were recruited in this retrospective study between March 2012 and December 2012. The baseline characteristics and information on sleep duration were collected by self-reported questionnaires. In addition, physical examination and blood test were also performed. The outcome events in this study were new onset of stroke, MI and tumors during subsequent follow-up. Multivariate logistic regressions were adopted to investigate the relationships between sleep duration and outcome events among different sleep duration groups (< 6 h, 6–7 h, 7–8 h [reference], 8–9 h, and > 9 h per day) in participants with MetS.

**Results:**

The mean self-reported total sleep duration was 7.8 ± 1.2 h. Compared with participants with MetS slept for 7–8 h per day, the adjusted odds ratios (ORs) for those slept for > 9 h in stroke, MI and tumors were 2.014 (95% confidence interval [CI]: 1.184–3.426, *P* = 0.010), 1.731 (95% CI: 0.896–3.344, *P* = 0.102) and 2.159 (95% CI: 0.991–4.704, *P* = 0.053), respectively, whereas the adjusted ORs for those slept for < 6 h in stroke, MI and tumors were 2.249 (95% CI: 0.973–5.195, *P* = 0.058), 1.213 (95% CI, 0.358–4.104, *P* = 0.756) and 1.743 (95% CI, 0.396–7.668, *P* = 0.462), respectively.

**Conclusions:**

Long sleep duration (> 9 h) significantly increased the risk of stroke but not MI and tumors in individuals with MetS compared with 7–8 h of sleep duration. Short sleep duration (< 6 h) was not associated with the increased risk of stroke, MI and tumors in individuals with MetS.

## Introduction

Metabolic syndrome (MetS) is a well-defined risk factor for cardiovascular disease (CVD) [1] and mortality [2]. Recently, sleep has been recognized as a modifiable contributor to MetS. Previous studies have reported a potential association between sleep duration and the development of MetS [3, 4], as well as each of its components, (i.e. diabetes [5], obesity [6], hypertension [7] and dyslipidemia [8]). Moreover, sleep duration was also found to be associated with CVD [9], stroke [10] and mortality [11].

Currently, evidence on the relationship of sleep duration with CVD and tumors in patients with MetS was still limited. A US study enrolled 1344 participants with MetS found that < 6 h sleep duration increased the risk of mortality caused by CVD and cerebrovascular diseases [12]. However, given a U-shape relationship usually existed in published epidemiological studies [13, 14], the effect of sleep duration as continuous type on varying health outcomes should be assessed. Hence it is hypothesized that long sleep duration (> 9 h per day) and short sleep duration (< 6 h per day) might increase the risk of stroke, myocardial infarction (MI) and tumors in individuals with MetS, compared with those slept for 7–8 h per day. Therefore, the current study was conducted to assess the association of sleep duration with stroke, MI, and tumors in a Chinese population with MetS.

## Methods

### Participants and public involvement

All participants have given written informed consent for authorizing to use their data. The study protocol was approved by the Committee on Human Research at Rui-Jin Hospital, School of Medicine, Shanghai Jiao Tong University (2014 clinical trial ethics approval No. 52).

### Study design

The participants in this retrospective study were from an ongoing longitudinal study (Risk Evaluation of cAncers in Chinese diabeTic Individuals, REACTION) that was designed to investigate the relationship between type 2 diabetes, prediabetes and the risk of cancer in Chinese population [15]. All permanent residents aged 35–75 year-old of the Jingding, Laoshan and Gucheng communities in Beijing, China were invited to participate by the primary health care centers. All the included participants lived in developed urban area and represent general middle-aged and elderly population of Beijing. A total of 19,314 persons were recruited between March 2012 and December 2012. Among them, 10,216 participants were followed up in 2015. In the current study, participants were additionally excluded as following: 1) self-reported sleep duration (< 4 h or > 12 h) due to too few participants; 2) indefinite diagnosis of MetS, stroke, MI, and tumors; and 3) substantial missing data.

Sample size calculations (PASS 11.0) were done based on a two­sided type I error of 5% and a power of 80%. Assuming an incidence of stroke was 3.5% in participants with MetS and the odds ratio (OR) for > 9 h of sleep duration versus 7–8 h was 1.5, 2052 participants would be required to identify the difference between the two groups. To compensate for losses to follow-up and to be powered for the primary endpoint assessment, the enrolment of a total of 2300 participants was planned.

### Questionnaire and data collection

Standardized questionnaires were used to collect baseline information, including self-reported sleep duration, medical history, physical exercise, depressive symptoms, smoking, and alcohol consumption. Total sleep duration of each individual was calculated from the time of sleeping to the time of waking-up. Participants were divided into five groups (< 6, 6–7 h, 7–8 h, 8–9 h, and > 9 h per day) based on their sleep duration, and 7–8 h of sleep duration was generally considered as the most appropriate sleep duration [16]. Depression symptoms were assessed using the Patient Health Questionnaire-9 (PHQ-9), which has been described in detail elsewhere [17]. Physical activity was defined as engaging in various sports more than once a week or engaging in heavy physical labor, such as steelmaking, agriculture, and casting. Smokers and drinkers were defined as participants who smoked one or more cigarettes per day and drank alcohol once or more a week for at least 6 months, respectively.

The height, weight, waist circumference and hip circumference for each participant were measured using anthropometric measurements. Body mass index (BMI) was calculated as body weight in kilograms divided by body height in meters squared (kg/m^2^). Blood pressure was measured three times at one-minute intervals using an Ohm electronic sphygmomanometer after seating for relax at least 5 min, and the average of three measurements was used for analysis.

### Blood test

Fasting blood samples were collected in the morning, and the level of triglycerides (TGs), total cholesterol (TC), low-density lipoprotein (LDL), high-density lipoprotein (HDL), fasting blood glucose (FBG), and postprandial blood glucose (PBG) were measured using an autoanalyser (Cobas 8000 modular analyzer series, Roche Diagnostics, Basel, Switzer land) (A.P.W and Y.X.W). Glycosylated hemoglobin A1c (HbA1c) was determined by high performance liquid chromatography using the VARIANT II Hemoglobin Testing System (Tosoh Corporation, Tokyo, Japan). Fasting and postprandial blood insulin levels were measured using the glucose oxidase-peroxidase method. Participants without a history of diabetes mellitus (DM) underwent a 75-g oral glucose tolerance test, while those with DM underwent a 100-g oral steam bread tolerance test.

### Metabolic syndrome

MetS was defined as having any 3 of the following 4 conditions according to the “Recommendations on metabolic syndrome from the Chinese Diabetes Association” [18]: 1) Overweight or obesity (BMI > 25 kg/m^2^); 2) abnormal glucose metabolism (FBG ≥ 6.1 mmol/L or PBG ≥ 7.8 mmol/L); 3) hypertension (systolic blood pressure [SBP] ≥ 140 mmHg and/or diastolic blood pressure [DBP] ≥ 90 mmHg, or a definite medical history of hypertension); and 4) abnormal lipid metabolism (TGs ≥ 1.7 mmol/L or HDL < 0.9 mmol/L (for males)/< 1.0 mmol/L (for females)).

### Outcomes

The outcome in the study were new onset stroke, MI and tumors during subsequent follow-up. The follow-up was performed via face to face visit or telephone visit. Participants were required to report the absence or presence of outcome events, and to provide information about the date of onset, whether and where they were hospitalized, clinical manifestations at the time of onset, and any clinical examination they underwent. The investigators (Y.N.Y and L.X.Z) would then go to these hospitals to review their medical records to ensure that data were correct.

### Statistical analysis

Continuous variables are presented as mean with standard deviation (SD) for those with normal distribution, and median with interquartile range (IQR, 25th - 75th) for those with skewed distributions. Categorical data are presented as count with proportions. One-way analysis of variance (ANOVA) and chi-square test were used as appropriate to test the differences in the general characteristics. Multivariate logistic regression was used to calculate the ORs for various outcome events in different sleep duration groups (< 6 h, 6–7 h, 7–8 h [reference], 8–9 h, and > 9 h). Three models were developed: Model 1 was not adjusted any covariates; Model 2 adjusted for age, sex and BMI; Model 3 adjusted for covariates in model 2 plus SBP, waist circumference, hip circumference, depression, blood glucose, triglycerides and physical activity. Statistical analysis was performed using SPSS software version 19.0 (Chicago, IL, USA). A two-sided *P* < 0.5 were considered statistically significance.

## Results

### Participant characteristics

A total of 8968 participants (3111 males and 5857 females), with a mean age of 56.7 ± 7.7 years old, were included in the study between March and December 2012 (Table 1 and Supplementary Fig. S1). The mean self-reported total sleep duration was 7.8 ± 1.2 h per day. The prevalence of overweight/obesity, hypertension, abnormal glucose metabolism and abnormal lipid metabolism were 57.0, 44.8, 47.0, and 33.2%, respectively. In all participants, those who slept for < 7 h or > 8 h per day were more likely to be overweight and depressive, and to have hypertension, abnormal glucose metabolism, abnormal lipid metabolism and MetS than those slept for 7–8 h per day. Significant differences were also observed between different sleep duration groups in age, BMI, waist circumference, hip circumference and TGs. The prevalence of stroke, MI and tumors at baseline was similar among different sleep duration groups.

**Table 1:** Baseline information according to self-reported total sleep duration among general participants

	ALL (*n* = 8968)	Sleep duration (h)					*P* value
		< 6 (*n* = 280)	6–7 (*n* = 950)	7–8 (*n* = 4785)	8–9 (*n* = 2114)	> 9 (*n* = 839)	
Age, years	56.7 ± 7.7	57.5 ± 7.4	56.4 ± 7.2	57.5 ± 8.8	57.3 ± 8.1	58.1 ± 8.6	< 0.001
Male, %	34.7 (3111)	35.0 (98)	31.9 (303)	34.4 (1648)	35.8 (756)	36.5 (306)	0.225
Waist circumference, cm	83.8 ± 8.9	84.9 ± 9.4	84.4 ± 9.2	86.0 ± 9.9	83.6 ± 8.7	84.1 ± 9.1	0.006
Hip circumference, cm	94.4 ± 6.9	95.4 ± 7.2	95.2 ± 7.0	97.0 ± 7.8	94.0 ± 6.7	93.8 ± 7.1	< 0.001
BMI, kg/m2	25.7 ± 3.4	26.0 ± 3.3	26.0 ± 3.5	24.7 ± 3.7	25.7 ± 3.4	25.8 ± 3.6	0.032
Stroke history, %	2.8 (250)	4.3 (12)	3.4 (32)	2.4 (113)	2.9 (62)	3.7 (31)	0.051
Myocardial infarction history, %	1.1 (102)	1.4 (4)	1.2 (11)	0.9 (42)	1.4 (30)	1.8 (15)	0.102
Tumor history, %	2.0 (183)	1.8 (5)	2.0 (19)	1.9 (89)	2.4 (50)	2.4 (20)	0.647
Smoking habits							0.606
Regular smoker, %	16.2 (1449)	16.4(46)	16.6 (158)	16.2 (777)	15.6 (330)	16.4 (138)	
Sometimes smoker, %	2.2 (197)	3.6 (10)	2.5 (24)	2.3 (110)	1.8 (39)	1.7 (14)	
Never smoker, %	81.6 (7322)	80.0 (224)	80.8 (768)	81.5 (3898)	82.5 (1745)	81.9 (687)	
Drinking habits							0.297
Regular drinker, %	10.2 (916)	11.1 (31)	11.6 (110)	9.7 (466)	10.1 (214)	11.3 (95)	
Sometimes drinker, %	18.9 (1698)	20.7 (58)	17.8 (169)	19.7 (945)	17.9 (379)	17.5 (147)	
Never drinker, %	70.9 (6354)	68.2 (191)	70.6 (671)	70.5 (3374)	71.9 (1521)	71.2 (597)	
Depression symptoms							< 0.001
Mild depression, %	5.6 (504)	12.1 (34)	7.6 (72)	4.9 (233)	5.2 (110)	6.6 (55)	
Moderate or severe depression, %	1.1 (97)	3.9 (11)	1.1 (10)	0.9 (42)	0.9 (18)	1.9 (16)	
Physical activity, %	9.8 (875)	10.7 (30)	8.8 (84)	10.2 (488)	9.1 (193)	9.5 (80)	0.518
Abnormal glucose metabolism, %	47.0 (4217)	47.1 (132)	46.1 (438)	44.9 (2149)	49.4 (1045)	54.0 (453)	< 0.001
Overweight (BMI ≥ 25), %	57.0 (5108)	65.0 (182)	58.6 (557)	55.8 (2672)	56.1 (1186)	60.9 (511)	0.002
Hypertension, %	44.8 (4019)	43.6 (122)	43.7 (415)	42.4 (2029)	48.5 (1026)	50.9 (427)	< 0.001
Abnormal lipid metabolism, %	33.2 (2974)	30.0 (84)	31.5 (299)	32.3 (1546)	34.0 (719)	38.9 (326)	0.002
Metabolic syndrome, %	30.7 (2754)	31.4 (88)	30.0 (285)	28.6 (1368)	32.6 (690)	38.5 (323)	< 0.001
HbA1c, %	5.8 (5.5, 6.3)	5.9 (5.5, 6.3)	5.9 (5.6, 6.4)	5.8 (5.5, 6.3)	5.9 (5.5, 6.3)	5.9 (5.5, 6.4)	0.002
SBP, mmHg	131.9 ± 17.7	131.6 ± 16.8	131.1 ± 15.8	131.9 ± 20.3	133.0 ± 18.8	133.9 ± 18.8	< 0.001
DBP, mmHh	75.9 ± 12.9	74.9 ± 8.9	75.6 ± 9.7	77.6 ± 10.9	76.2 ± 15.0	75.7 ± 10.1	0.452
TGs, mmol/L	1.33 (0.97, 1.87)	1.27 (0.97, 1.82)	1.28 (0.96, 1.83)	1.33 (0.97, 1.88)	1.36 (0.96, 1.90)	1.36 (0.99, 1.88)	0.006
TC, mmol/L	5.23 ± 1.00	5.19 ± 0.89	5.24 ± 0.98	5.12 ± 1.13	5.21 ± 0.95	5.27 ± 1.05	0.585
HDL, mmol/L	1.44 ± 1.63	1.44 ± 0.38	1.44 ± 0.37	1.33 ± 0.34	1.40 ± 0.35	1.40 ± 0.34	0.555
LDL, mmol/L	3.20 ± 0.81	3.14 ± 0.76	3.20 ± 0.81	3.02 ± 0.89	3.20 ± 0.78	3.23 ± 0.87	0.493
FBG, mmol/L	5.32 (4.93, 6.03)	5.32 (4.93, 6.00)	5.32 (4.92, 6.05)	5.28 (4.91, 5.96)	5.37 (4.94, 6.10)	5.41 (5.01, 6.27)	0.001
PBG, mmol/L	7.98 (6.46, 10.66)	8.18 (6.42, 10.93)	7.89 (6.46, 10.51)	7.92 (6.45, 10.50)	8.15 (6.49, 11.03)	8.20 (6.51, 10.96)	< 0.001

Data were presented % (n), mean (standard deviation), or median (25–75 percentile)

Abbreviation: *BMI* Body mass index; *HbA1c* Glycosylated hemoglobin A1c; *SBP* Systolic blood pressure; *DBP* Diastolic blood pressure; *TGs* Triglycerides; *TC* Total cholesterol; *HDL* High density lipoprotein; *LDL* Low density lipoprotein; *FBG* Fasting blood glucose; *PBG* Postprandial blood glucose

MetS at baseline was observed in 2752 (30.7%) participants. In participant with MetS, there were no significant differences between different sleep duration groups in blood glucose, blood lipid and BMI (Table 2). Only hypertension, one component of MetS, was significantly associated with sleep duration. Additionally, there were significant differences between different sleep duration groups in age, alcohol consumption, hip circumference, and depression among these sleep duration groups.

**Table 2:** Baseline information according to self-reported total sleep duration among participants with metabolic syndrome

	Sleep duration (h)					*P* value
	< 6 (*n* = 88)	6–7 (*n* = 285)	7–8 (*n* = 1368)	8–9 (*n* = 690)	> 9 (*n* = 323)	
Age, years	59.0 ± 7.8	58.1 ± 7.3	57.8 ± 7.4	59.3 ± 8.2	60.0 ± 8.5	< 0.001
Male, %	47.7 (42)	35.1 (100)	38.5 (527)	39.3 (271)	42.4 (137)	0.041
Waist circumference, cm	90.7 ± 7.7	89.8 ± 7.8	89.4 ± 7.7	88.8 ± 7.6	89.8 ± 6.8	0.097
Hip circumference, cm	97.9 ± 7.0	98.1 ± 7.1	97.7 ± 6.7	96.8 ± 6.7	97.0 ± 6.7	0.016
BMI, kg/m^2^	27.8 ± 2.6	28.2 ± 3.2	28.0 ± 2.8	27.9 ± 3.0	28.0 ± 2.6	0.729
Stroke history, %	9.1 (8)	3.9 (11)	3.5 (48)	4.5 (31)	5.3 (17)	0.090
Myocardial infarction history, %	3.4 (3)	1.4 (4)	1.8 (25)	2.2 (15)	3.1 (10)	0.476
Tumor history, %	1.1 (1)	2.1 (6)	1.9 (26)	3.0 (21)	3.1 (10)	0.391
Smoking habits						0.088
Regular smoker, %	20.5 (18)	16.8 (48)	16.2 (222)	14.3 (99)	19.2 (62)	
Sometimes smoker, %	5.7 (5)	2.8 (8)	2.4 (33)	2.6 (18)	0.6 (2)	
Never smoker, %	73.9 (65)	80.4 (229)	81.4 (1113)	83.0 (573)	80.2 (259)	
Drinking habits						0.047
Regular drinker, %	15.9 (14)	11.9 (34)	10.3 (141)	9.4 (65)	13.6 (44)	
Sometimes drinker, %	22.7 (20)	18.2 (52)	20.2 (276)	17.2 (119)	13.9 (45)	
Never drinker, %	61.4 (14)	69.8 (199)	69.5 (951)	73.3 (506)	72.4 (234)	
Depression symptoms						< 0.001
Mild depression, %	6.9 (6)	6.7 (19)	5.3 (72)	6.2 (43)	6.8 (22)	
Moderate or severe depression, %	0 (0)	1.1 (3)	0.5 (7)	0.6 (4)	4.0 (13)	
Physical activity, %	8.0 (7)	8.4 (24)	9.4 (128)	8.6 (59)	8.7 (28)	0.957
Abnormal glucose metabolism, %	89.8 (79)	86.7 (247)	85.2 (203)	85.7 (591)	87.0 (281)	0.705
Overweight (BMI ≥ 25), %	94.3 (83)	93.3 (266)	92.0 (1258)	90.1 (622)	94.4 (305)	0.128
Hypertension, %	73.9 (65)	82.5 (235)	80.6 (1103)	84.5 (583)	85.8 (277)	0.019
Abnormal lipid metabolism, %	63.6 (56)	66.7 (190)	69.2 (946)	70.0 (483)	69.3 (224)	0.688
HbA1c, %	6.3 (5.9, 7.2)	6.1 (5.7, 7.0)	6.1 (5.8, 6.7)	6.2 (5.8, 6.7)	6.2 (5.9, 6.9)	0.493
SBP, mmHg	140.0 ± 15.8	140.2 ± 15.8	140.7 ± 18.0	142.0 ± 19.4	143.9 ± 18.4	0.033
DBP, mmHh	78.4 ± 9.6	79.3 ± 10.0	80.1 ± 15.7	80.2 ± 18.9	79.2 ± 10.4	0.674
TG, mmol/L	1.89 (1.29, 2.48)	1.90 (1.40, 2.45)	1.95 (1.43, 2.56)	1.90 (1.42, 2.59)	1.96 (1.52, 2.52)	0.401
TC, mmol/L	5.29 ± 1.04	5.39 ± 1.02	5.35 ± 1.06	5.29 ± 1.06	5.40 ± 1.05	0.485
HDL, mmol/L	1.24 ± 0.29	1.28 ± 0.32	1.36 ± 4.06	1.23 ± 0.29	1.25 ± 0.27	0.872
LDL, mmol/L	3.27 ± 0.88	3.34 ± 0.83	3.31 ± 0.89	3.28 ± 0.87	3.34 ± 0.88	0.821
FBG, mmol/L	6.23 (5.50, 7.45)	6.07 (5.46, 7.29)	6.02 (5.42, 6.95)	6.10 (5.51, 7.03)	6.24 (5.61, 7.35)	0.200
PBG, mmol/L	9.72 (8.28, 12.60)	9.34 (7.96, 12.95)	9.26 (7.91, 12.17)	9.43 (7.90, 12.63)	9.57 (7.90, 12.44)	0.565

Data were presented % (n), mean (standard deviation), or median (25–75 percentile)

Abbreviation: *BMI* Body mass index; *HbA1c* Glycosylated hemoglobin A1c; *SBP* Systolic blood pressure; *DBP* Diastolic blood pressure; *TGs* Triglycerides; *TC* Total cholesterol; *HDL* High density lipoprotein; *LDL* Low density lipoprotein; *FBG* Fasting blood glucose; *PBG* Postprandial blood glucose

### The association between sleep duration and MetS

**Fig. 1:**
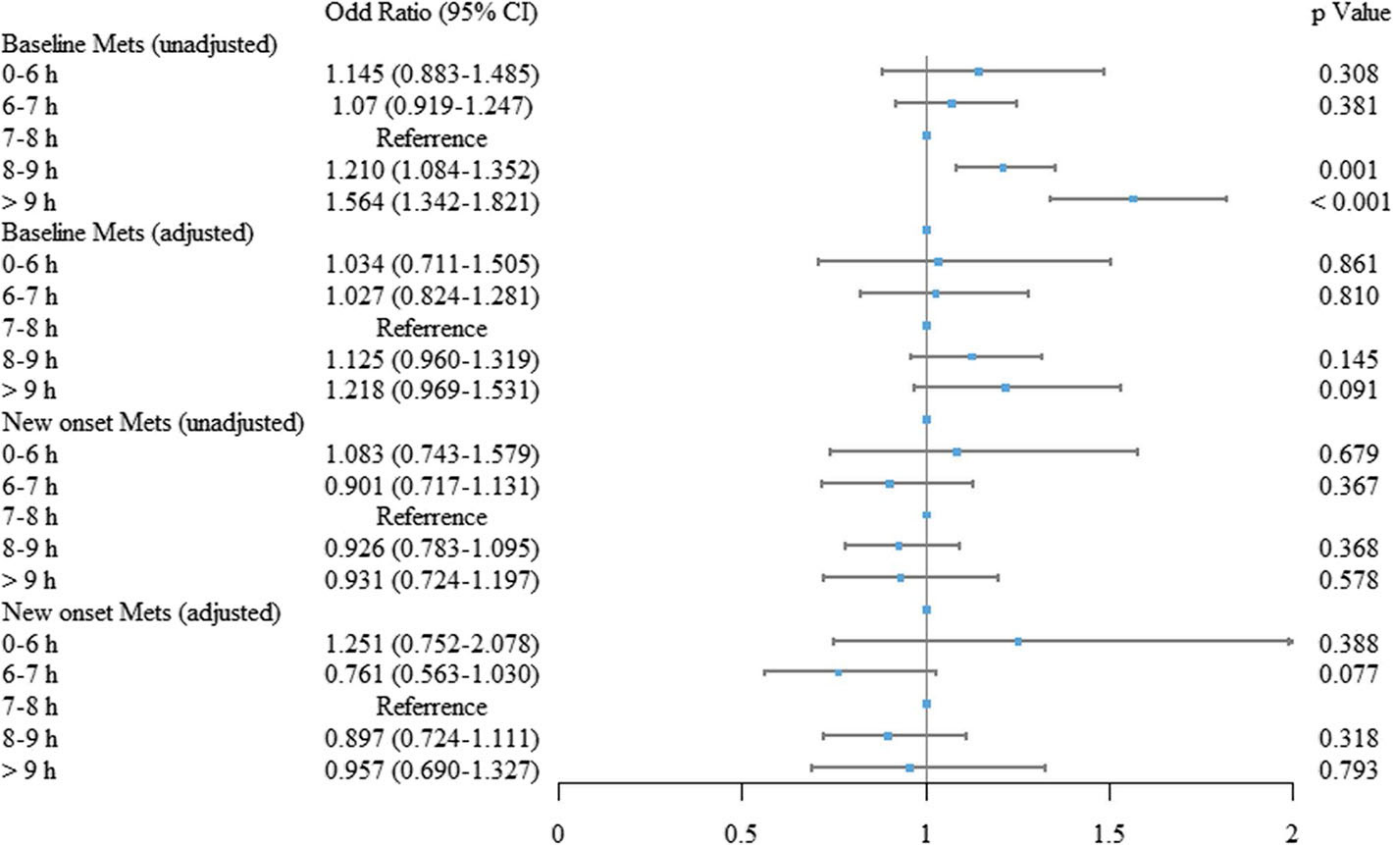
The relationship between sleep duration and metabolic syndrome. The forest plot summarised the ORs (95% CI) of different sleep durations for baseline or new onset metabolic syndrome

At baseline, compared to participants who slept for 7–8 h per day, the ORs for MetS in those who slept for < 6 h, 6–7 h, 8–9 h and > 9 h were 1.145 (95% confidence interval [CI]: 0.883–1.485, *P* = 0.308), 1.070 (95% CI: 0.919–1.247, *P* = 0.381), 1.210 (95% CI: 1.084–1.352, *P* = 0.001) and 1.564 (95% CI: 1.342–1.821, *P* < 0.001), respectively. After adjusting for age, sex, SBP, BMI, depression, blood glucose, TGs and physical activity, the ORs for MetS of individuals who slept for < 6 h, 6–7 h, 8–9 h and > 9 h were 1.034 (95% CI: 0.711–1.505, *P* = 0.861), 1.027 (95% CI: 0.824–1.281, *P* = 0.810), 1.125 (95% CI: 0.960–1.319, *P* = 0.145) and 1.218 (95% CI: 0.969–1.531, *P* = 0.091), respectively (Fig. 1).

At follow-up, 1033 new onset MetS were observed. Compared with individuals who slept for 7–8 h per day, the risk of new onset MetS did not differ significantly for those who slept for < 6 h (OR: 1.251; 95% CI: 0.752–2.078; *P* = 0.388), 6–7 h (OR: 0.761; 95% CI: 0.563–1.030; *P* = 0.077), 8–9 h (OR: 0.897; 95% CI: 0.724–1.111; *P* = 0.318), and > 9 h (OR: 0.957; 95% CI: 0.690–1.327; *P* = 0.793), respectively (Fig. 1).

### The association between sleep duration and outcome events

**Fig. 2:**
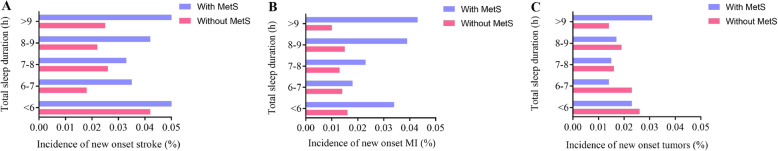
The incidence rates of new onset stroke (**a**), new onset MI (**b**), and new onset tumors (**c**) and during follow-up stratified by sleep duration. Each bar represents the percentage of new onset outcome events in < 6 h, 6–7 h, 7–8 h, 8–9 h and > 9 h sleep duration

During follow-up, 267 of stroke, 162 of MI and 159 of tumors were identified, respectively. In participants with baseline MetS, the number of events was 114 in stroke, 81 in MI and 49 in tumors (Fig. 2). Compared with participants with MetS slept for 7–8 h per day, the adjusted ORs for those slept for > 9 h in stroke, MI and tumors were 2.014 (95% CI: 1.184–3.426, *P* = 0.010), 1.731 (95% CI: 0.896–3.344, *P* = 0.102) and 2.159 (95% CI: 0.991–4.704, *P* = 0.053), respectively (Table 3), whereas the adjusted ORs for those slept < 6 h for stroke, MI and tumors were 2.249 (95% CI: 0.973–5.195, *P* = 0.058), 1.213 (95% CI: 0.358–4.104, *P* = 0.756) and 1.743 (95% CI: 0.396–7.668, *P* = 0.462), respectively (Table 3). Moreover, the incidence of stroke had a near U-shape curve relationship with sleep duration (Fig. 2). For participants without MetS, the risks of stroke, MI and tumors were not significantly different between the different sleep duration groups.

**Table 3:** Risk for health outcomes according to total sleep duration

	Tumor				Myocardial infarction				Stroke			
	Without MetS		With MetS		Without MetS		With MetS		Without MetS		With MetS	
	OR	*P* value	OR	*P* value	OR	*P* value	OR	*P* value	OR	*P* value	OR	*P* value
Model 1												
Total sleep duration, h												
< 6	1.605 (0.635, 4.053)	0.317	1.492 (0.344, 6.466)	0.593	1.245 (0.383, 4.051)	0.715	1.474 (0.442, 4.910)	0.528	1.645 (0.786, 3.443)	0.187	2.541 (1.111, 5.811)	0.027
6–7	1.385 (0.779, 2.464)	0.268	0.913 (0.311, 2.680)	0.869	1.077 (0.522, 2.219)	0.842	0.746 (0.288, 1.930)	0.545	0.695 (0.378, 1.278)	0.242	1.069 (0.532, 2.147)	0.852
7–8	1		1		1		1		1		1	
8–9	1.160 (0.730, 1.844)	0.530	1.135 (0.555, 2.321)	0.728	1.174 (0.694, 1.986)	0.549	1.700 (1.010, 2.861)	0.046	0.870 (0.577, 1.310)	0.504	1.290 (0.801, 2.076)	0.295
> 9	0.825 (0.374, 1.821)	0.635	2.049 (0.955, 4.395)	0.065	0.768 (0.303, 1.947)	0.578	1.892 (0.997, 3.588)	0.051	0.978 (0.542, 1.764)	0.940	2.254 (1.343, 3.783)	0.002
Model 2												
Total sleep duration, h												
< 6	1.567 (0.620, 3.963)	0.342	1.563 (0.360, 6.797)	0.551	1.261 (0.385, 4.124)	0.702	1.281 (0.382, 4.292)	0.688	1.586 (0.754, 3.337)	0.224	2.368 (1.031, 5.435)	0.042
6–7	1.379 (0.775, 2.453)	0.275	0.911 (0.310, 2.677)	0.866	1.111 (0.537, 2.296)	0.777	0.746 (0.287, 1.935)	0.546	0.703 (0.381, 1.294)	0.257	1.067 (0.531, 2.146)	0.855
7–8	1		1		1		1		1		1	
8–9	1.153 (0.725, 1.834)	0.548	1.161 (0.567, 2.379)	0.683	1.063 (0.625, 1.806)	0.822	1.573 (0.930, 2.660)	0.091	0.802 (0.531, 1.212)	0.295	1.231 (0.763, 1.986)	0.395
> 9	0.814 (0.368, 1.797)	0.610	2.135 (0.991, 4.599)	0.053	0.666 (0.261, 1.699)	0.395	1.671 (0.875, 3.193)	0.120	0.864 (0.477, 1.567)	0.630	2.097 (1.244, 3.535)	0.005
Model 3												
Total sleep duration, h												
< 6	1.377 (0.536, 3.535)	0.506	1.743 (0.396, 7.668)	0.462	1.135 (0.342, 3.762)	0.836	1.213 (0.358, 4.104)	0.756	1.421 (0.669, 3.017)	0.361	2.249 (0.973, 5.195)	0.058
6–7	1.339 (0.750, 2.389)	0.324	0.927 (0.313, 2.742)	0.891	1.009 (0.485, 2.098)	0.981	0.745 (0.286, 1.942)	0.547	0.669 (0.362, 1.237)	0.200	1.013 (0.503, 2.043)	0.970
7–8	1		1		1		1		1		1	
8–9	1.162 (0.730, 1.850)	0.527	1.142 (0.555, 2.347)	0.718	1.067 (0.625, 1.820)	0.812	1.677 (0.984, 2.855)	0.057	0.799 (0.529, 1.209)	0.288	1.228 (0.759, 1.988)	0.403
> 9	0.810 (0.366, 1.791)	0.602	2.159 (0.991, 4.702)	0.053	0.675 (0.264, 1.727)	0.412	1.731 (0.896, 3.344)	0.102	0.883 (0.487, 1.602)	0.683	2.014 (1.184, 3.426)	0.010

Model 1 was not adjusted any covariates

Model 2 adjusted for age, sex and BMI

Model 3 adjusted for covariates in model 2 plus SBP, depression, blood glucose, triglycerides and physical activity

## Discussion

The present study investigated the associations between sleep duration and the occurrence of stroke, MI and tumors in a Chinese population with MetS. The findings suggested that compared to participants who slept for 7–8 h per day, those slept for > 9 h had a significantly increased risk of stroke but a similar risk of MI and tumors, while those slept for < 6 h had a similar risk of stroke, MI and tumors.

The current study investigated the associations between sleep duration and stroke, MI and tumors, which are closely related to the survival, health status and quality of life of population. Previous study [12] had reported that < 6 h of sleep duration increased the risk of MetS related mortality. The current study added evidence that > 9 h of sleep duration was also associated with adverse events in MetS patients. However, significant association was only identified between > 9 h of sleep duration and stroke, other results just showed a trend towards association but did not reach statistical significance. The results were possibly ascribed to that not only the limited sample size but also the small numbers of subjects with outcome events. A second follow-up of these participants is currently underway, and the findings will be updated and further validated in future studies. Even though, these findings also suggest that sleep management might play an important role in patients with MetS.

The association between sleep and the prognosis of MetS might be partially explained as following. First, participants with worse sleep habits could also have higher cardiovascular risk, indicated by higher prevalence of obesity, depression, hypertension, abnormal glucose metabolism, and abnormal lipid metabolism. Second, inadequate sleep durations can result in systemic inflammation. Long sleep duration (> 8 h) are associated with subclinical inflammation and increased arterial stiffness [19, 20], while sleep curtailment increases proinflammatory cytokine [21], high-sensitivity C-reactive protein [22], and white blood cell [23] levels, which causes endothelial dysfunction and promotes the incidence of vascular complications of diabetes [24]. Third, changes in the sympathetic nervous system by long or short sleep durations may cause high levels of catecholamines and increase blood pressure and heart rate [25]. Sleep regulates the activity of the hypothalamic pituitary adrenal (HPA) axis, which was reduced during sleep onset and the early stages of sleep, and was activated during the later stages of sleep [26, 27]. Therefore, short sleep durations may weaken the inhibitory effect of early-stage sleep on the HPA axis. Conversely, long sleep durations may enhance the activation effect of rapid eye movement stage on the HPA axis, thereby maintaining the activity of the HPA axis at a higher level, which has adverse effects on metabolic health [27].

### Study strengths and limitations

The strengths of the present study is its cohort design and the relatively large number of participants, which enabled us to investigate the causal relationship of sleep duration and the health outcomes. In addition, information on a wide range of potential confounders/modifiers and their potential effects were taken into account. The present study also has several limitations to acknowledge. First, the study was limited by retrospective nature. Second, objective information on obstructive sleep apnea (OSA), a cardiovascular risk factor [28], was not available and thus not considered in this study. However, the use of objective methods (like polysomnography) to accurately measure the OSA is usually not feasible in large studies of general populations, which is a common limitation of sleep-related epidemiological studies [9]. Third, the sample size might fail to reach statistical power for some outcomes (MI and tumors) and unable to well represent Chinese population, so the results should be cautiously generalized. Forth, sleep duration was determined according to a self-reported questionnaire, this parameter was not measured objectively although similar to many prior epidemiological studies.

## Conclusions

The results showed that long sleep duration (> 9 h) significantly increased the risk of stroke but not MI and tumors in individuals with MetS compared with 7–8 h of sleep duration. Short sleep duration (< 6 h) was not associated with the increased risk of stroke, MI and tumors in individuals with MetS. These findings revealed the relationships of sleep duration with stroke in a Chinese population with MetS. A near U-shape curve was observed between sleep duration and incident stroke. Future longitudinal studies with large samples are warranted to verify the health effect of sleep duration for patients with Mets.

## Supplementary information

**Additional file 1: Fig. S1**. Flowchart of participant selection

**Additional file 2.** CONSORT 2010 Checklist

## Data Availability

Additional data are available from the corresponding author for reasonable requesting.

## Abbreviations

MetS: Metabolic syndrome
CVD: Cardiovascular disease
MI: Myocardial infarction
REACTION: Risk evaluation of cancers in Chinese diabetic individuals
OR: Odds ratio
PHQ-9: Patient health questionnaire-9
BMI: Body mass index
TGs: Triglycerides
TC: Total cholesterol
LDL: Low-density lipoprotein
HDL: High-density lipoprotein
FBG: Fasting blood glucose
PBG: Postprandial blood glucose
HbA1c: Glycosylated hemoglobin A1c
DM: Diabetes mellitus
SBP: Systolic blood pressure
DBP: Diastolic blood pressure
SD: Standard deviation
IQR: Interquartile range
ANOVA: Analysis of variance
CI: Confidence interval
HPA: Hypothalamic pituitary adrenal
OSA: Obstructive sleep apnea
