# Supplementary material for: Association of sleep duration with stroke, myocardial infarction, and tumors in a Chinese population with metabolic syndrome: a retrospective study
Source: Lipids Health Dis. 2020 Jun 27;19:155. doi: 10.1186/s12944-020-01328-1 (PMC7321539; doi:10.1186/s12944-020-01328-1)
Supplement: Supplementary file 1 — Additional file 1: Fig. S1. Flowchart of participant selection [file 12944_2020_1328_MOESM1_ESM.docx]

8,968 participants included

Assessed for eligibility on baseline (n = 19,314 )

Participants were followed up in 2015 (n = 10,216)

Lost to follow-up (n = 9,098)

1,248 participants were excluded due to:

- <4 h or >12 h sleep duration (n = 776);
- indefinite diagnosis of new onset stroke, MI and tumors (n = 294);
- substantial missing data (n = 178).

Fig. S1. Flowchart of participant selection
